# Protocols in Cleft Lip and Palate Treatment: Systematic Review

**DOI:** 10.1155/2012/562892

**Published:** 2012-11-01

**Authors:** Pedro Ribeiro Soares de Ladeira, Nivaldo Alonso

**Affiliations:** ^1^School of Medicine, University of São Paulo, São Paulo, SP, Brazil; ^2^Division of Burns and Plastic Surgery, Department of Surgery, School of Medicine, University of São Paulo, São Paulo, SP, Brazil; ^3^Rua Afonso Brás, 473 cj 65 Vila Nova Conceição, 04511-000 São Paulo, SP, Brazil

## Abstract

*Objectives*. To find clinical decisions on cleft treatment based on randomized controlled trials (RCTs). *Method*. Searches were made in PubMed, Embase, and Cochrane Library on cleft lip and/or palate. From the 170 articles found in the searches, 28 were considered adequate to guide clinical practice. *Results*. A scarce number of RCTs were found approaching cleft treatment. The experimental clinical approaches analyzed in the 28 articles were infant orthopedics, rectal acetaminophen, palatal block with bupivacaine, infraorbital nerve block with bupivacaine, osteogenesis distraction, intravenous dexamethasone sodium phosphate, and alveoloplasty with bone morphogenetic protein-2 (BMP-2). *Conclusions*. Few randomized controlled trials were found approaching cleft treatment, and fewer related to surgical repair of this deformity. So there is a need for more multicenter collaborations, mainly on surgical area, to reduce the variety of treatment modalities and to ensure that the cleft patient receives an evidence-based clinical practice.

## 1. Introduction

Orofacial clefts are the most prevalent craniofacial birth defects and the second most common birth anomaly, second only to clubfoot [1]. In the United States of America, it is estimated that $100,000 are spent to rehabilitate a child born with oral cleft [2]. 

The approach of the patient with cleft lip and palate is multidisciplinary, and the cleft team should be ideally composed by craniofacial surgeons, otolaryngologists, geneticists, anesthesiologists, speech-language pathologists, nutritionists, orthodontists, prosthodontists, and psychologists, and to be capable of treating even rare facial clefts with excellence, neurosurgeons, and ophthalmologists. In this manner, it is possible to provide long-term followup through the entire child's development and achieve all of the following treatment goals: normalized facial aesthetic, integrity of the primary and secondary palate, normal speech and hearing, airway patency, class I occlusion with normal masticatory function, good dental and periodontal health, and normal psychosocial development [3]. 

The most broadcast treatment modalities in the management of unilateral cleft lip and palate are listed in Table 1 (chronologic age) and Table 2 (dentofacial development).

The presented management of cleft lip and palate is not accepted exactly by all cleft centers, and there is a striking diversity of clinical practice in the area [4]. Evidence-based medicine should be the answer to the uncertainties in the treatment; however there is a paucity of high level of evidence (i.d. systematic reviews and randomized controlled trials [5]) on cleft lip and palate [6]. Therefore, many clinical decisions are made based on biased evidence, such as the decision of when to perform secondary bone graft, which is answered by many surgeons with the information of a retrospective study [7]. 

Aiming to find clinical decisions based on randomized controlled trials (RCTs), searches on cleft lip and/or palate were done in three main scientific databases: Cochrane Library, Embase, and PubMed [8]. Posteriorly, we selected articles that could validate or change the presented management. 

## 2. Methods

On March 3, 2012, searches for RCTs were made in three databases (Cohrane Library, Embase, and PubMed) on cleft lip and/or palate. When appropriate, we used search strategies involving the MeSH descriptors and Emtree, Boolean logic operators, and free-text truncated with an asterisk. 

The main descriptors used were as follows:

- MeSH: “cleft lip,” “cleft palate”;
- Emtree: “cleft lip palate,” “cleft lip,” “cleft palate.”

### 2.1. Cochrane Library

The searches in this database were made in “Search History,” and the search strategy was assembled in “Search For.”

We used MeSH descriptors when available, and free-text truncated with an asterisk. The following expression was added to the search strategy: “AND (randomized controlled trial*):ti,ab,kw.” Only the results in “Cochrane Central Register of Controlled Trials” were considered. 

### 2.2. Embase

The searches in this database were made in “Advanced Search,” selecting the following items: “Map to preferred terminology (with spell check),” “Also search as free text,” and “Include sub-terms/derivatives.” In “Records from,” we selected only “Embase.” 

We used Emtree descriptors and selected the item “Randomized Controlled Trial” in “Advanced Limits,” option “Evidence Based Medicine.” 

### 2.3. PubMed

The searches in this database were made in “Search details.”

We used MeSH descriptors when available, and free-text truncated with an asterisk. Additionally, we selected “Randomized Controlled Trial” in “Limits,” option “Type of Article.”

**Figure 1:**
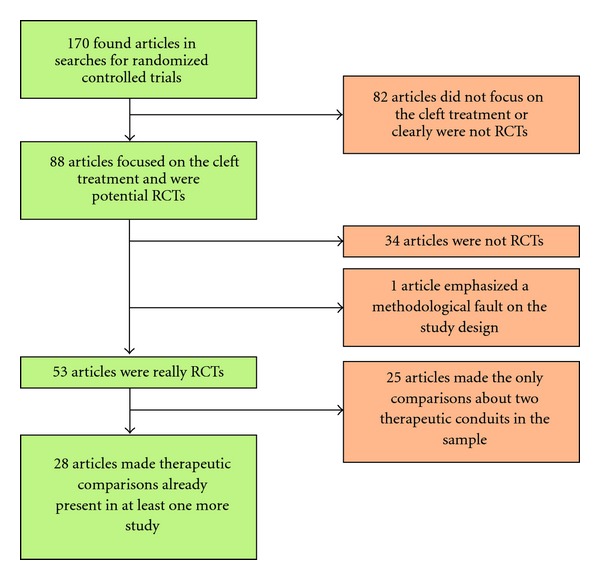
Flowchart outlining the selection process of the 28 articles.

All abstracts provided by the databases in the searches were collected, resulting in a total of 170 different articles. From these abstracts, studies that clearly were not RCTs (e.g., reviews and case series) or not focused on cleft treatment were excluded. As a result, we were left with 88 papers. Posteriorly, we searched “Portal de Periódicos da CAPES” (http://www.capes.gov.br/) for the full-text articles. After meticulous reading of the studies, we verified that 53 of the 88 articles were really RCTs. Next, the approaches compared in each study were analyzed, in order to select articles in which the comparisons discussed appeared in two or more of the 53 studies. At the end of the selection, 28 articles were included for the analysis of the obtained conclusions. One paper on infant orthopedics was excluded since it emphasized a methodological fault on the study design (i.d. sample heterogeneity). The flowchart below (Figure 1) outlines the process of articles selection. 

## 3. Results

**Figure 2:**
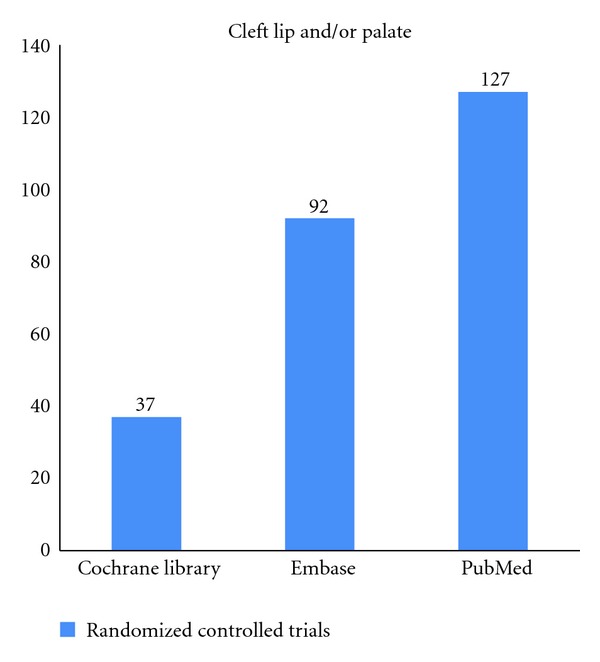
Results of searches on “Cleft Lip and/or Palate” in the three databases.

The search results were recorded in Figure 2.

The issues discussed by the 28 selected articles were as follows:

- infant orthopedics: 15/28 = 53.57%;
- postoperative pain relief: 5/28 = 17.86%;
- management of the cleft maxillary hypoplasia: 4/28 = 14.28%;
- perioperative steroids: 2/28 = 7.14%;
- alveoloplasty: 2/28 = 7.14%.

The conclusions of the 28 papers are presented by issue addressed in Tables 3, 4, 5, 6, and 7, where the signs “<,” “>,” and “=” mean, respectively, that experimental approach is “less recommended than,” “more recommended than,” and “equivalent to” control.

## 4. Discussion

 RCTs and systematic reviews of these studies are considered the best levels of evidence to conduct clinical practice [49]. For this reason, we searched for RCTs that could orientate the cleft lip and/or palate treatment, which is marked by a great number of approaches [10]. To find the RCTs we used two main databases proposed by World Health Organization (i.d. PubMed and Embase) [8] and the Cochrane Library, reference of studies for evidence-based medicine practice [50]. Therefore, from the 170 articles found in the searches for RCTs, we reached 28 final studies that approached therapeutic practices on cleft patient. The issues of these 28 articles were collected together with their conclusions.

 During this study, a scarce number of RCTs were found approaching cleft treatment. This fact is confirmed by the literature, which showed in 2004 that only 6% of RCTs in “Plastic and Reconstructive Surgery,” “British Journal of Plastic Surgery,” and “Annals of Plastic Surgery” approached cleft lip and/or palate [51]. In an article of 2007, it is possible to observe that the lack of studies with high level of evidence is a problem present in the whole plastic surgery, resulting in the following distribution of articles from the 16 leading journals in the area: case report, 80%; RCTs, 2%; and meta-analysis, <1% [52]. 

 In the final sample of 28 articles, we verified the presence of five issues, arranged here in descending order by number of studies that addressed them: infant orthopedics, postoperative pain relief, management of the cleft maxillary hypoplasia, and perioperative steroids and alveoloplasty. The issues of the selected articles are consistent with the importance given by the literature, because of the following: the infant orthopedics efficacy is debatable since its creation, nearly six decades ago [53, 54]; postoperative pain relief for children has become a necessary practice recently, about two decades ago, on account of several myths on pediatric pain and lack of scientific knowledge in the area [55]; the question whether to use or not osteogenesis distraction instead of the conventional Le Fort I osteotomy on cleft maxillary hypoplasia divides opinions in the scientific community [56, 57]; perioperative steroids are a common practice in craniofacial surgery; however it has several documented side effects [58, 59] and few well designed studies [46]; alveoloplasty is a highly debated intervention, especially with the advent of bone substitutes [60].

 From the five issues, only two approached the surgical act, resulting in 21.43% (6/28) of the selected articles. This fact reflects the lack of RCTs on surgical procedures itself. A survey of 2003 supports this affirmation, which estimates that only 3.4% of the publications on the main surgical journals were RCTs [61]. 

From the final sample, 53.57% were composed by articles approaching infant orthopedics. That was due to the fact that 14 of the 15 studies on this issue were part of the Dutch Intercenter Study (Dutchcleft), a great effort to analyze the effects of presurgical infant orthopedic treatment in complete unilateral cleft lip and palate [32]. 

 No study in the selected sample analyzed specifically a cleft patient with a syndrome or congenital abnormality. That explains the difficulty of treating orofacial clefts related to over 300 syndromes [62], and the twenty percent of all children with a cleft that have other congenital abnormality, part of a known syndrome or not [63]. 

 From the 15 studies approaching infant orthopedics, only 3 found a benefit of this treatment, an improvement of the patient speech at nearly 2.5 years old [33–35]. However, when the language development was evaluated in the long term, at nearly 6 years old, there were no differences between experimental group and control [31]. On the other hand, all of the 14 Dutchcleft studies used only the Zurich approach to treat their patients whereas the other articles applied a passive and an active maxillary orthopedic treatment. So, in our systematic review we did not find RCTs about nasoalveolar molding therapy, a promising practice in presurgical infant orthopedics [54]. Non-RCTs studies have been appointing to the benefits of nasoalveolar molding therapy: long-term aesthetic outcomes [64–66] and better nasal symmetry [67].

 Four distinct comparisons were found on postoperative pain relief: rectal acetaminophen versus placebo, bilateral nerve block with bupivacaine versus plain saline, continuous bupivacaine infusion through iliac crest catheter versus plain saline, and intravenous ketorolac with morphine versus morphine alone. There was a divergence between the conclusions of the two RCTs found on the first comparison; one appointed an effective pain control and equivalence in regards to nauseas and vomits (rectal loading dose of 40 mg/kg followed by 30 mg/kg 8 hourly) [36], whereas the other one did not observe effective pain control (single prophylactic dose of 40 mg/kg) [37], emphasizing the fact that both analyzed palatoplasties. These results are in concordance with the standard clinical practice that states a postoperative rectal loading dose (30–40 mg/kg) followed by regular maintenance doses (20 mg/kg 6 hourly or 30 mg/kg 8 hourly) [68–70]. Besides the difficulties associated with rectal administration (e.g., delayed and erratic absorption), an RCT done in patients undergoing craniosynostosis repair verified a higher efficacy than oral administration [68].

 On the comparison of bilateral nerve block with bupivacaine versus plain saline, there were one article approaching palatal block [38] and two approaching infraorbital nerve block [39, 40]. Prabhu et al. proved with a randomized, double blind, prospective clinical trial that bilateral infraorbital nerve block with bupivacaine is more effective than peri-incisional infiltration in postoperative pain relief after cleft lip repair [71], and it became a standard clinical practice in cleft care [72, 73]. However, the scientific literature does not clarify which volume should be used to make this block, so there is evidence defending 0.5 mL of 0.5% bupivacaine in each side [74], 3 mL of 0.5% bupivacaine [75], and 0.5–1 ml of 0.5% bupivacaine [73]. Since very small doses of bupivacaine have serious side effects, such as cardiac dysrhythmias and neurotoxicity [76], more studies need to be done to standardize this technique. In regards to the article that analyzed palatal block, there were no differences between experimental group (0.5 mL of 0.25% bupivacaine at each point) and control (0.5 mL of plain saline at each point), with both resulting in postoperative analgesia. Besides the extensive use of palatal block [77], including in Smile Train [78], the results of the selected RCT led us to conclude that the analgesic effects are results from the liquid pressure, not from the anesthetic solution. Such results appeared in a similar manner with Van Gheluwe and Walton, explaining the analgesic effect of intrapulpal saline injection with the pressure that it exerts [79]. 

 In regards to the studies that addressed the management of cleft maxillary hypoplasia [41–44] there was only one comparison, distraction osteogenesis versus Le Fort I osteotomy. There were no divergences in the studies conclusions, leading to a possible superiority of distraction osteogenesis over the conventional technique. However, the scientific literature induces us to conclude that the choice between distraction osteogenesis and conventional orthognathic surgery is dependent on advancement length. Baek et al. published a controlled clinical trial comparing these techniques in which they realize this fact [80]. Scolozzi, in a review of 80 scientific articles, concludes that distraction osteogenesis should be applied for displacements larger than 6 mm in cleft patients [81]. On the other hand, as a meta-analysis on the issue concludes, there is little high-level evidence to safely guide the surgeon in this decision [82]. 

 On the issue “perioperative steroids,” both selected articles [45, 46] conclude in favor of intravenous dexamethasone sodium phosphate perioperative administration in palatoplasties. Since there is a substantial risk of postoperative airway obstruction after palatoplasties (one of the most common postoperative problems) [83] and other craniofacial surgeries [84], perioperative steroids became a standard practice in many craniofacial centers [45, 84, 85]. However these two RCTs did not report relevant side effects; the samples were too small (45 [45] and 20 [46] patients) to analyze, without a high bias, steroids complications, such as psychosis [58, 59] and hypertension [86].

The use of bone substitutes is one way to avoid the morbidities associated with performance of autogenous bone graft in alveoloplasties, and one of the most promising is the bone morphogenetic protein-2 (BMP-2) [87]. The two RCTs found on alveoloplasties [47, 48] concluded that BMP-2 is superior to the conventional technique, with an increase in bone regeneration and reduced patient morbidity. However, since the samples used were small (16 [48] and 21 [47] patients), we still can not assure its safety in relation to theoretical risks: non-small-cell lung cancer; pancreatic and oral cancer; heterotopic ossification and undesirable bone growth, even in the malignant form [87]. The lack of larger studies has been highlighted by a Cochrane review on the issue [60].

 This systematic review found RCTs in favor of the following: rectal acetaminophen 40 mg/kg administered in the operating room at the end of the palatoplasty, and 30 mg/kg every 8 hours until 48 hours; bilateral infraorbital nerve block with bupivacaine in children who will undergo cleft lip repair; osteogenesis distraction in the cleft maxillary hypoplasia treatment; intravenous dexamethasone sodium phosphate 0.25 mg/kg before palatoplasty and every 8 hours (two doses after surgery); alveoloplasty with BMP-2. However, a far from ideal number of non-Dutchcleft RCTs (14 articles) proved to be of high quality, which can be verified by the following parameters [88]: 57.14% (8/14) explicit the randomization mode; 21.43% (3/14) report allocation concealment; 21.43% (3/14) made clear how they calculated the sample size. In regards to Dutchcleft studies, all of them explicit in some way the randomization mode and allocation concealment. These high-quality RCTs proved that infant orthopedics with Zurich approach is not recommended to the cleft patient. More collaborations such as Dutchcleft need to be done to safely guide cleft teams around the world, and to decrease the huge variety of practices in this area in the long term. 

## 5. Conclusion

Few randomized controlled trials were found approaching cleft treatment, and fewer related to surgical repair of this deformity. From the found articles, only a small percentage reported the study with known quality parameters. However, one consistent conclusion could be verified due to fourteen Ducthcleft RCTs; there is no high-level evidence supporting the use of infant orthopedics by Zurich approach. So there is a need for more multicenter collaborations, mainly on surgical area, to reduce the variety of treatment modalities and to ensure that the cleft patient receives an evidence-based clinical practice.

## Figures and Tables

**Table 1:** Treatment modalities in the management of unilateral cleft lip and palate which are often based on chronologic age.

Timing	Procedure
After 16 weeks of pregnancy	Cleft lip diagnosis by ultrasound images (palate is more difficult to acquire) [9]
Prenatal	Discussion with a craniofacial surgeon [10]
	Consultation with a geneticist/dysmorphologist [10]
Neonatal	If the child has cleft palate, specialized nipples and bottles are necessary to improve feeding after birth [11]
12 weeks of age	Cleft lip repair [12]
6–12 months of age	Cleft palate one-stage repair with intravelar veloplasty [13]
5 years	Secondary rhinoplasty [14]

**Table 2:** Treatment modalities in the management of unilateral cleft lip and palate which are often based on dentofacial development.

Timing	Procedure
Prior to cleft lip repair	Presurgical infant orthopedics [15]
Primary dentition	Orthodontic treatment for maxillary expansion [16]
Mixed dentition	Orthodontic treatment for maxillary expansion and maxillary protraction [16]
Before eruption of permanent dentition	Secondary alveolar bone graft with cancellous bone from iliac crest [10, 17]
Permanent dentition	Orthodontic treatment for dental arches alignment [18]
After fully eruption of permanent dentition, dental arches alignment, and end of the maxillofacial growth	Orthognathic surgery for maxillary advancement [16]
After orthognathic surgery	Postsurgical orthodontics for closure of residual spaces and occlusion final adjustments [19]
	Replacement of missing teeth by a prosthodontist [20]

**Table 3:** Conclusions of articles that addressed “infant orthopedics”.

Infant Orthopedics			
Experimental group	Conclusion	Control	Explanation for the conclusion
Patients who had infant orthopedics	=	Patients who did not have infant orthopedics	Cephalometric outcomes at ages 4 and 6 were not relevant [21]; no long-term (age 6 [22]) or short-term (18 months [23]) outcomes on facial appearance; no influence on mother's satisfaction [24]; no improvement on feeding efficiency or general body growth within the first year [25, 26]; no long-term outcomes on the maxillary arch dimensions (age 6 [27]), on deciduous dentition (age 6 [27, 28]), or on the occlusion (age 6 [28]); no short-term outcomes on the maxillary arch dimensions (18 months) [29, 30]; no long-term outcomes on language development (age 6) [31]; no improvement on the intelligibility at 2.5 years [32].

Patients who had infant orthopedics	>	Patients who did not have infant orthopedics	Acceptable cost effectiveness based on speech development at 2.5 years [33]; better speech development between 2 and 3 years [34]; higher ratings for intelligibility at 2.5 years [35].

**Table 4:** Conclusions of articles that addressed “postoperative pain relief”.

Postoperative pain relief*			
Experimental group	Conclusion	Control	Explanation for the conclusion
Rectal Acetaminophen	>	Rectal placebo	In children who underwent palatoplasty, acetaminophen (40 mg/kg administered in the operating room at the end of surgery, and 30 mg/kg every 8 hours until 48 hours) was more effective in pain control than placebo [36].

Rectal Acetaminophen (40 mg/kg)	=	Rectal placebo	Acetaminophen and placebo were equivalents in regards to nauseas and vomits, the most frequent adverse effects [36]. Rectal acetaminophen (administered at anesthesia induction) did not result in analgesic plasma concentrations and it was not effective in pain control after palatoplasties [37].

Bilateral Palatal Block with Bupivacaine (0.5 mL of 0.25% solution at greater palatine, lesser palatine, and nasopalatine foramina)	=	Plain saline (0.5 mL at each point)	Bupivacaine and saline were effective in the palatal block and provided good parental satisfaction. Both provided better postoperative analgesia than the no block group [38].

Bilateral Infraorbital Nerve Block with Bupivacaine	>	Plain saline	In children who underwent cleft lip repair, the injection of 1.5 mL of 0.25% bupivacaine (extra-oral approach) [39] or 1–1.5 mL of 0.5% bupivacaine (intraoral approach) [40] in the area of infraorbital foramen provided safe and prolonged postoperative pain relief (at least 8 hours [39]).

*All the alveoloplasties used iliac crest bone graft.

**Table 5:** Conclusions of articles that addressed “management of the cleft maxillary hypoplasia”.

Management of the cleft maxillary hypoplasia			
Experimental group	Conclusion	Control	Explanation for the conclusion
Osteogenesis distraction	=	Le Fort I osteotomy	No significant differences were found in development of velopharyngeal insufficiency postoperatively [41, 42] and patient morbidity (infection and occlusion disturbance) [43].

Osteogenesis distraction	>	Le Fort I osteotomy	Better skeletal stability in maintaining the maxillary advancement in the long term [43, 44].

**Table 6:** Conclusions of articles that addressed “perioperative steroids”.

Perioperative steroids			
Experimental group	Conclusion	Control	Explanation for the conclusion
Intravenous dexamethasone sodium phosphate	>	Intravenous dextrose solution	In patients who underwent primary palatoplasty, intravenous dexamethasone sodium phosphate 0.25 mg/kg before surgery and every 8 hours (two doses after surgery) lowered the risks of postoperative airway distress and fever [45], and no adverse sequelae were verified [46].

**Table 7:** Conclusions of articles that addressed “alveoloplasty”.

Alveoloplasty			
Experimental group	Conclusion	Control	Explanation for the conclusion
Cleft repair with BMP-2 (bone morphogenetic protein-2)	>	Iliac crest bone graft	Increased bone regeneration and lower patient morbidity: oral wound quality, pain, infection, paresthesia, and donor area wound healing [47]; infection, paresthesia, neuropathy, and donor area wound healing [48].
